# Dynamics of Neuromuscular Transmission Reproduced by Calcium-Dependent and Reversible Serial Transitions in the Vesicle Fusion Complex

**DOI:** 10.3389/fnsyn.2021.785361

**Published:** 2022-02-15

**Authors:** Alejandro Martínez-Valencia, Guillermo Ramírez-Santiago, Francisco F. De-Miguel

**Affiliations:** ^1^Posgrado en Ciencias Físicas, Universidad Nacional Autónoma de México, Ciudad de México, Mexico; ^2^Instituto de Fisiología Celular-Neurociencias, Universidad Nacional Autónoma de México, Ciudad de México, Mexico; ^3^Instituto de Matemáticas, Universidad Nacional Autónoma de México, Juriquilla, Mexico

**Keywords:** transmitter release, neuromuscular synapse, facilitation, depression, synapse, fusion complex, kinetics, calcium

## Abstract

Neuromuscular transmission, from spontaneous release to facilitation and depression, was accurately reproduced by a mechanistic kinetic model of sequential maturation transitions in the molecular fusion complex. The model incorporates three predictions. First, calcium-dependent forward transitions take vesicles from docked to preprimed to primed states, followed by fusion. Second, prepriming and priming are reversible. Third, fusion and recycling are unidirectional. The model was fed with experimental data from previous studies, whereas the backward (β) and recycling (ρ) rate constant values were fitted. Classical experiments were successfully reproduced with four transition states in the model when every forward (α) rate constant had the same value, and both backward rate constants were 50–100 times larger. Such disproportion originated an abruptly decreasing gradient of resting vesicles from docked to primed states. By contrast, a three-state version of the model failed to reproduce the dynamics of transmission by using the same set of parameters. Simulations predict the following: (1) Spontaneous release reflects primed to fusion spontaneous transitions. (2) Calcium elevations synchronize the series of forward transitions that lead to fusion. (3) Facilitation reflects a transient increase of priming following the calcium-dependent maturation transitions. (4) The calcium sensors that produce facilitation are those that evoke the transitions form docked to primed states. (5) Backward transitions and recycling restore the resting state. (6) Depression reflects backward transitions and slow recycling after intense release. Altogether, our results predict that fusion is produced by one calcium sensor, whereas the modulation of the number of vesicles that fuse depends on the calcium sensors that promote the early transition states. Such finely tuned kinetics offers a mechanism for collective non-linear transitional adaptations of a homogeneous vesicle pool to the ever-changing pattern of electrical activity in the neuromuscular junction.

## Introduction

In the present study, we searched for a unifying molecular mechanism by which neuromuscular transmission adapts dynamically to the ongoing pattern of electrical activity. Four aspects of transmission were analyzed in detail. (1) Spontaneous release at rest (Fatt and Katz, 1952), (2) calcium dependence evoked release on an impulse (Katz and Miledi, 1979), (3) facilitation, namely a non-linear increase of release upon rapid subsequent stimulation (Feng, 1940; Eccles et al., 1941; Liley and North, 1953; del Castillo and Katz, 1954b; Katz and Miledi, 1968), and (4) depression, namely a reduction of release on stimulation at extended intervals under high release probability (Eccles et al., 1941; Feng, 1941; Lundberg and Quilisch, 1953; del Castillo and Katz, 1954b; Takeuchi, 1958; Betz, 1970).

Understanding release requires a collective analysis of the events regulating vesicle fusion. An essential study by del Castillo and Katz (1956) showed that release may occur from any active zone region of presynaptic terminals. It is also well-accepted that vesicle fusion requires a mature, commonly named “primed” molecular assembly [for review see Becherer and Rettig (2006), Sudhof (2013), Neher and Brose (2018), Gandini and Zamponi (2021)]. Maturation of the fusion complex follows a stereotyped sequence of molecular transitions that will be resumed here as follows: (1) docking (*D*) is the early tethering of vesicles with the plasma membrane upon establishment of boundaries between vesicle, membrane, and soluble proteins; (2) prepriming (*pP*) occurs upon stabilization of the molecular complex; and (3) priming (*P*) occurs when vesicles become competent for fusion. Fusion (*F*) is evoked by calcium activation of the primed complex, mediated by the vesicle protein synaptotagmin. That only a small (∼1–3%) fraction of the vesicle pool fuses on an impulse (Fatt and Katz, 1952; Katz and Miledi, 1979) has suggested that most vesicles rest in immature docked or preprimed states. After fusion, vesicles are recycled and resupplied from a large “reserve pool” of non-tethered vesicles to a new docked state (*F → D*; del Castillo and Katz, 1956; Heuser and Reese, 1973; Betz and Angleson, 1998; Dulubova et al., 2005; Andrews-Zwilling et al., 2006; Kittel et al., 2006; Sudhof, 2013; Weimer et al., 2006; Imig et al., 2014; Gan and Watanabe, 2018; Neher and Brose, 2018).

**FIGURE 1:**
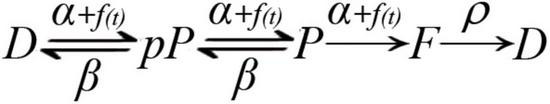
Kinetic model of molecular transitions of the fusion complex in individual vesicles. *D*, docked; *pP*, preprimed; *P*, primed; *F*, fusion; α, forward rate constant; *f(*t*)*, calcium time dependence of the forward transition; β, backward rate constant; ρ, recycling rate constant. *D ⇌ pP ⇌ P* are bidirectional; *P* → *F* → *D* are unidirectional; spontaneous transitions occur following the corresponding rate constant. On electrical activity, the calcium-dependence accelerates the *Dp* ⇀ *pP* ⇀ *P* ⇀ *F* transitions.

Based on the molecular transitions that determine the amount of vesicles ready for release and on the calcium-dependence of some such transitions (Gingrich and Byrne, 1985; Worden et al., 1997; Burgoyne, 2007; Hosoi et al., 2007; Neher and Sakaba, 2008; Craxton, 2010; Corbalán-García and Gómez-Fernández, 2014; Burgoyne et al., 2019), we put forward the hypothesis schematized in Figure 1, according to which the dynamic adaptations in the number of vesicles that fuse upon variations in nerve activity express a calcium-dependent, collective, and reversible maturation of the fusion complex.

Our hypothesis considers that the *D ⇌ pP ⇌ P* transitions are bidirectional, with characteristic forward (α) and backward (β) rate constants. The α values are similar for all transitions; both β values are also similar but different from α. Reversibility is supported in the neuromuscular junction from electron tomography observations of vesicles that change their dynamic equilibrium from docked to previous states (Jung et al., 2016), and from experiments and modeling of preprimed to primed transitions in crayfish neuromuscular junction (Pan and Zucker, 2009). In addition, ribbon synapses display continuous docking and undocking of vesicles (Zenisek et al., 2000). On an action potential, calcium evokes fusion and promotes further maturation of fusion complexes. Rapid arrival of a subsequent impulse evokes facilitation. Backward transitions gradually reduce facilitation and return vesicles to their resting levels. After intense release, depression is produced by slow vesicle recycling (Otsuka et al., 1962; Glavinović and Narahashi, 1988; Delgado et al., 2000), aided by the reversible transitions of primed vesicles predicted here.

The experimental exploration of our hypothesis exceeds the current technical possibilities. However, mathematical modeling provides a solid alternative (Gingrich and Byrne, 1985; Varela et al., 1997; Worden et al., 1997; Dittman et al., 2000; Shahrezaei et al., 2006; Pan and Zucker, 2009; Dittrich et al., 2013; Herman and Rosenmund, 2015; Neher, 2015). Here, we used a master equation based on the Gillespie (1976) stochastic algorithm to simulate the sequence of maturation transitions shown in Figure 1. Each vesicle with its fusion complex is a unit of a large homogeneous pool that responds collectively to each presynaptic impulse. The model was fed with experimental data from the literature. Undetermined parameters were fitted for convincing reproduction of well-known experiments of neuromuscular transmission in frog or cat. The code used for the simulations in this study is freely available at: https://github.com/alexini-mv/kinetic-neurotransmission.

## Results

Spontaneous and evoked presynaptic vesicle fusion were accurately reproduced by a sequence of four maturation kinetic states in the vesicle fusion complex. The condition was that all forward transitions had the same α value and were calcium-dependent, whereas the backward transitions had a β value 50–100 times larger than α. A three-state model failed to reproduce all forms of transmission with a single set of parameters. By contrast, five or six sequential kinetic steps reproduced all forms of release tested and provided a proportional increase in α and a reduction in β. The parameters that reproduced cat and frog neuromuscular transmission are shown in Table 1.

**TABLE 1:** Kinetic parameters that reproduce neuromuscular transmission in frog and cat.

Preparation	Kinetic transitions	α (s^–1^)	β (s^–1^)	λ (*β/α*)	ρ (s^–1^)
Cat	4	0.62*	62	100	1.0
Frog	4	0.3	15.0	50	1.0
Frog	5	0.62	13.0	21	1.0
Frog	6	1.43	9.5	13	1.0

*From Boyd and Martin (1956a).

### Spontaneous Quantal Release

**FIGURE 2:**
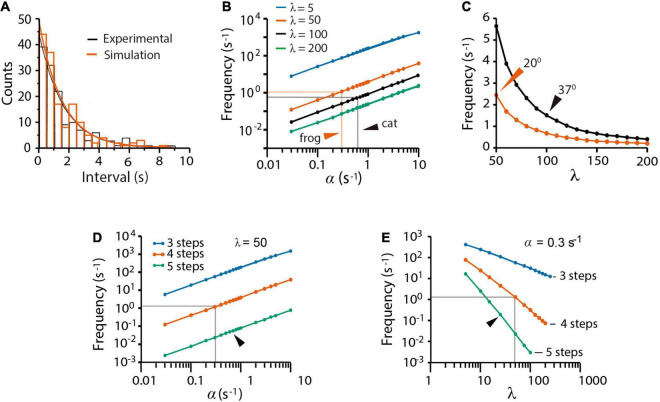
Spontaneous quantal release. **(A)** Experimental (black) and simulated (red) time distributions of spontaneous mepp_*s*_ from 5-min recording intervals. The experimental distribution of 143 mepp_*s*_ was obtained with license from Boyd and Martin (1956b); the simulation contains 148 mepp_*s*_. The 1.54 s decay half time of the experimental probability rendered the value of α as 0.62 s^–1^ value used in simulations of cat neuromuscular transmission along the paper. **(B)** Predicted contributions of the α and λ values on the mepp spontaneous frequency. Arrowheads point to values that gave the best fittings in simulations of frog and cat transmission. **(C)** Predicted mepp_*s*_ frequency as a function of the λ coefficient in frog (vermillion) and cat (black) synapses. The ρ = 1.0 value was equally successful in all simulations. Arrowheads point to experimental mepp frequencies at the indicated temperatures, from Boyd and Martin (1956a). **(D)** Effect of α on release with different number of kinetic steps. **(E)** Effect of λ on release with different number of kinetic steps. The gray lines in **(D,E)** are the α and λ values that reproduce all forms of release in frog neuromuscular junction. Arrowheads indicate the α and λ values that reproduce release with five kinetic steps. Three kinetic steps failed to reproduce spontaneous release regardless of the α and λ values.

The spontaneous quantal release in cat presynaptic neuromuscular terminals, reported by Boyd and Martin (1956a), was fairly reproduced by our model fed with an α = 0.62 s^–1^ value, obtained as the inverse of the experimental 1.61 s time constant (τ) of the time interval distribution of miniature end plate potentials (mepp_*s*_). An unexpectedly large β = 100α (λ = β/α = 100 coefficient) and a ρ = 1.0 s^–1^ recycling rate constant contributed to produce 148 ± 2 mepp_*s*_ at a 1.40 ± 0.10 s^–1^ frequency (*n* = 250 simulations), quite similar to the 143 mepp_*s*_ recorded at a 1.43 ± 0.88 s^–1^ frequency in the original study (Figure 2A). The experimental distribution of the intervals between mepp_*s*_ was fitted by the function *n* = *n*_*T*_(△*t*/τ)*e*^−*t*/_τ_^ (Fatt and Katz, 1952), where *n_T_* is the number of quanta released and △*t* = 0.5s is the bin size.

The mepp_*s*_ frequency (Figure 2B) was proportional to α and inversely proportional to β. Our best explanation to this result was that the large β value keeps a reduced pool of primed vesicles, therefore, reducing the probability of spontaneous fusion. Figure 2C compares simulations of cat and frog spontaneous release. The value of β = 0.62 s^–1^ (λ = 100) that reproduced the 1.43 s^–1^ mepp frequency in cat recordings at 37°C (Boyd and Martin, 1956a) quadruples the β = 15 s^–1^ (λ = 50) coefficient that reproduced the 2.5 s^–1^ mepp frequency commonly recorded from frog synapses at 20°C (see Fatt and Katz (1952)). The larger rate constant values in mammalian neuromuscular synapses may reflect the characteristic higher physiological temperature of mammalian tissues.

The previous results may be explained in the following way. First, spontaneous release reflects spontaneous *P* → *F* transitions, and second, the small probability of spontaneous release depends on the large λ coefficient, which maintains a small pool of primed vesicles at rest. Since a majority of experimental evidence used here proceeds from experiments in frog, the simulations that follow used the α = 0.3 and β = 50 values, unless otherwise indicated.

#### Kinetic Steps Contributing to Spontaneous Release

Figures 2D,E shows that a three-step version of the model failed to reproduce spontaneous release. Data in Figure 2D predicts that each kinetic step reduces the α-dependence of spontaneous release by more than one logarithmic unit. Since the frequency of spontaneous release (Figure 2D) depends on the number of primed vesicles, the three-state sequence with conventional α and β values must contain ∼3,000 primed vesicles, corresponding to 30% of the total pool of tethered vesicles. In such a situation, a fast train of three impulses would suffice to deplete the pool. It will be confirmed in the following sections that we did not find a set of variables capable to reproduce all forms of transmission with the tree-step version of the model. By contrast, a five-step version of the model reproduced spontaneous release provided an increase of α and a reduction of λ (Figure 2E). Therefore, a four-step *D ⇌ pP ⇌ P → F → D* transition cycle is necessary and sufficient to explain spontaneous release.

#### Calcium and Evoked Release

A useful experimental strategy to study statistical fluctuations of quantal release consists of reducing the extracellular calcium concentration and adding extracellular magnesium (del Castillo and Katz, 1954a; Boyd and Martin, 1955). Under such conditions, the number of quanta released by presynaptic impulses is drastically reduced and can be precisely predicted by the Poisson distribution (del Castillo and Katz, 1954a; Boyd and Martin, 1955). The theory states that the probability “*p*” of releasing “*x*” number of quanta (*x* = 0, 1, 2, 3, …, *n*) in a series of trials is low, whereas the number “*n*” of vesicles in the pool remains large. Even when *p* and *n* are experimentally elusive, the product *m* = *pn*, which is the average number of quanta released per impulse is measurable from the recordings and provides a direct means for the calculations.

**FIGURE 3:**
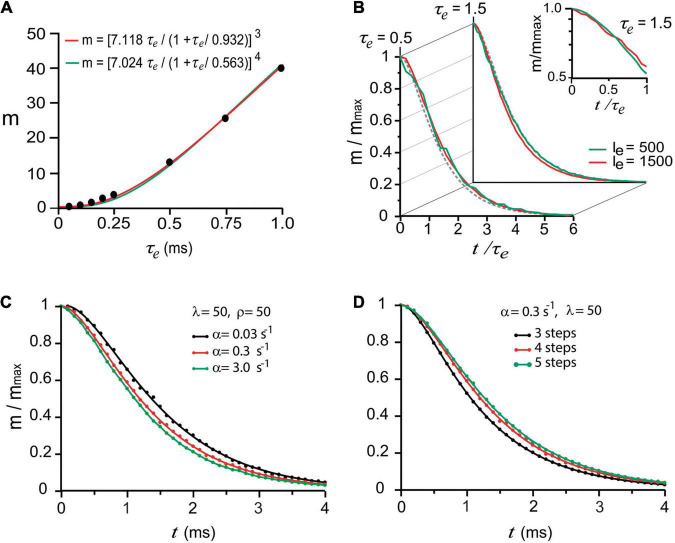
“Calcium-dependence” of quantal release. **(A)** The mean number of quanta (m) depends on the mean decay time (τ_*e*_) of the intracellular calcium increase. The dots are model predictions; the lines were obtained with the equation by Dodge and Rahamimoff (1967) with third and fourth order cooperativities. **(B)** The normalized number of quanta (m/m_max_) depends on the normalized *t/τ_*e*_* duration of the calcium signal. The traces are superimpositions of curves obtained using two different amplitudes (I_*e*_, arbitrary units) of calcium signal. The semilogarithmic chart in the inset shows the dispersion from a single exponential behavior below *t/τ_*e*_* = 1. **(C)** Increasing the value of α accelerated the release. **(D)** Adding kinetic steps to the model increased the latency of release.

To reproduce such experimental observations, stimulation impulses were coupled to an artificial calcium elevation whose amplitude and duration were adjusted to evoke the release of small numbers of quanta (see methods). The hypothesis that nerve impulses induce forward transitions in each maturation transition was tested by coupling the calcium transient to every α rate constant. Based on the observation by Katz and Miledi (1968, 1979) that the amount of release increases with the duration of depolarization, i.e., with the duration and amount of calcium entry, we adjusted the decay time (τ_*e*_) of the artificial calcium transient as a way to control the amount of release. With such approximation, the *m* value was increased in proportion to τ_*e*_. The simulations in Figure 3A reproduced the experimental calcium-dependence according to the equation by Dodge and Rahamimoff (1967); see also Smith et al. (1985), Augustine and Charlton (1986), expressing third (*R*^2^ = 0.999) or fourth order (*R*^2^ = 0.998) cooperativities, in our case, as the τ_*e*_ of the calcium elevation is increased. This approach has the advantage that increasing the τ_*e*_ value increases release and facilitation (Katz and Miledi, 1968; Gingrich and Byrne, 1985), and reducing the τ_*e*_ value reproduces the effect of addition of intracellular calcium buffers on release and facilitation (Kamiya and Zucker, 1994).

The next question was if one stimulus impulse may produce fusion of vesicles that rested in the *D* of *pP* states upon excitation. Exponential decays are conventional biophysical reporters of the fusion of multiple vesicles (Fatt and Katz, 1952). However, in the case of sequential two-state transitions, the collective output is expected to be described as the sum of two exponentials, the second of which reports the transitions that anticipate fusion. This hypothesis was tested in a series of trials simulating release under high release probability by using either a long τ_*e*_ value or different calcium transient amplitudes. The normalized number of quanta $\left(\frac{m}{m_{max}}\right)$, as a function of the normalized time (*t*/τ_*e*_) in Figure 3B, displayed similar exponential decays in the form *m*/*m*_*max*_ = (1 + *A*)*e*^−*t*/τ_*e*_^−*Ae*^−*t*/*x*τ_*e*_^, regardless of the τ_*e*_ or transient amplitude values. As shown in Figures 3B,C, the second exponential, which appeared when the evaluation time was briefer than τ_*e*_ originates from the combined contribution of α (Figure 3C) and the number of kinetic steps in the model (Figure 3D). The major elongation of the latency for release in Figure 3D occurred when the sequence of transitions had four instead of three steps, indicating that fusion of vesicles originally in *pP* state contributed to release. The major elongation of the latency for release in Figure 3D occurred when the sequence of transitions had four instead of three steps, indicating that fusion of vesicles originally in *pP* state contributed to the release. An additional elongation of the latency for release on the addition of another step to the sequence is an indicator of a smaller contribution of vesicles that rested in *D* state. The lack of effect of β and ρ is attributed to the recovery of the vesicle pool between subsequent stimulation pulses.

#### Evoked Quantal Release Under Low Probability

**FIGURE 4:**
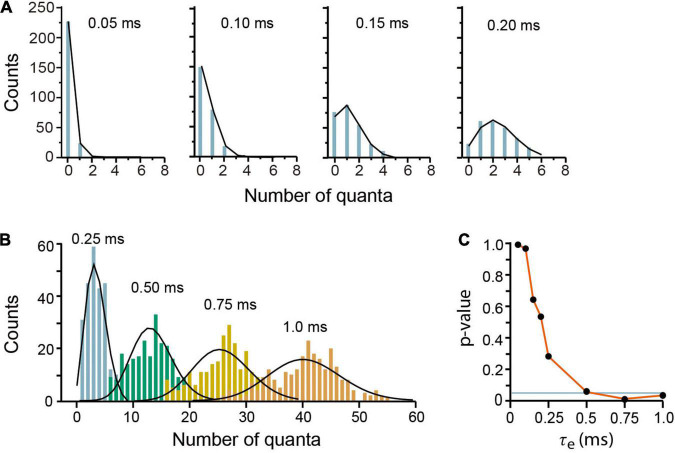
Evoked quantal release at low probability experimental conditions. **(A)** Amplitude distributions of quantal release in frog neuromuscular junction. Counts are the number of quanta from single runs of the program; the black lines link the discrete Poisson classes. The τ_*e*_ values are above in each plot. **(B)** Amplitude distributions at increasing probabilities by use of larger τ_*e*_ values. The discrepancies between the simulations and the Poisson predictions are clear with τ_*e*_ values above 0.25 ms. Each plot contains data from 250 stimuli mediated by a 5-s recovery interval. **(C)** Pearson’s significance (p) dependence on the τ_*e*_ value. The horizontal line indicates the 0.05 significance.

Our model reproduced convincingly quantal release under low release probability in frog neuromuscular junction (del Castillo and Katz, 1954a). Brief 0.05–0.15 ms τ_*e*_ values produced mepp_*s*_ amplitude distributions, with two (τ_*e*_ = 0.05 ms) to five (τ_*e*_ = 0.15 ms) amplitude classes including failures (Figure 4A). The Poisson equation reproduced such distributions when τ_*e*_ ≤ 0.5 (Pearson *x*^2^ > 0.05 coefficients). Larger τ_*e*_ values produced a reduction in the number of failures and an increase in the number of classes in the distribution. Values of τ_*e*_ greater than 0.25 deviated the amplitude distributions from the Poisson predictions (Figures 4B,C), as in experimental observations made under higher release probability (Boyd and Martin, 1956b).

### The Backward Rate Constant Influences the Release Probability

**FIGURE 5:**
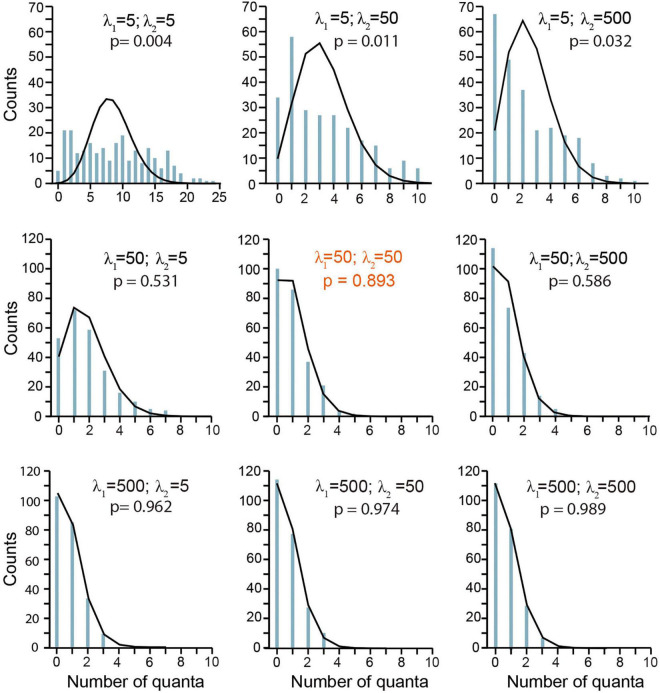
Contribution of the backward rate constant to quantal release. Data are presented in terms of the λ = β/α coefficients. λ_1_ corresponds to *D ⇌ pP*; λ_2_ corresponds to *pP ⇌ P*. The plots are as in Figure 4. The Pearson’s significance (p) appears in each chart. The central chart was obtained with λ_1_ = λ_2_ = 50, which fitted every form of release in frog synapses. Other parameters were α = 0.3 s^–1^, ρ = 1.0 s^–1^, and τ_*e*_ = 0.15 ms.

Simulations of frog experiments made under low probability conditions (del Castillo and Katz, 1954a; Katz and Miledi, 1968) allowed a further analysis on the contribution of β to quantal release. The λ coefficients of the *D ⇌ pP* (λ_1_) and *pP ⇌ P* (λ_2_) transitions were varied independently, whereas the α = 0.3 s^–1^, ρ = 1.0, and τ_*e*_ = 0.15 ms remained fixed. The λ_1_ = λ_2_ = 50 values reproduced transmission, as seen in the central chart of Figure 5.

The value of λ_1_ markedly influenced the number of quanta discharged per impulse. A small λ_1_ = 5 (β = 5α; top panels in Figure 5) that decelerates the *D* ↼ *pP* transition extended the range of classes in the distribution, which deviated from the predictions of the Poisson equation (*p* ≤ 0.05). Even the largest λ_2_ = 500 value tested failed to compensate for the effect of a reduced λ_1_. By contrast, a large λ_1_ = 500 value constrained the amplitude mepp distribution to a small-class range that was predicted by the Poisson distribution, regardless of λ_2_ (bottom plots in Figure 5). However, it will be shown below that this result only applies to release on single impulses as the large λ_1_ = 500 values failed to reproduce short-term plasticity. In spite of that, the results in this section underscore the essential contribution of the backward *D* ↼ *pP* transition to maintain a small resting pool of primed vesicles.

#### Facilitation and Depression

The effects of presynaptic stimulation under high release probability conditions can be studied by blocking acetylcholine receptors with curare to evoke only subthreshold postsynaptic responses (del Castillo and Katz, 1956; Betz, 1970). In such conditions, a stimulation train gradually induces facilitation to turn into depression, presumably owing to a reduction of the releasable vesicle pool (Otsuka et al., 1962; Mallart and Martin, 1968; Betz, 1970). This section reproduces the experimental transition from facilitation to depression in frog neuromuscular junction. The experimental protocol was a conditioning train of three impulses, followed by a test impulse 250 ms later (Mallart and Martin, 1968). A long τ_*e*_ = 1.3 ms simulated the effect of residual calcium in experiments by Katz and Miledi (1968), who obtained facilitation by elongating calcium entry. This manipulation allowed to simulate the elimination of residual calcium by reducing the τ_*e*_ value of the third conditioning impulse.

**FIGURE 6:**
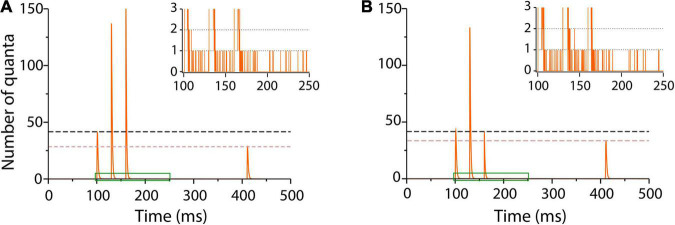
Sequence of facilitation and depression in frog neuromuscular junction. **(A)** Number of quanta released in response to a train of three conditioning pulses 30 ms apart, followed by a test pulse 250 ms later (Mallart and Martin, 1968). Facilitation on the second and third impulses was followed by depression on the test pulse. The traces are averages of 1,000 runs in the program. The inset amplifies a single run in the region contained in green to show asynchronous release after the conditioning impulses. The simulation parameters were α = 0.3 s^–1^; λ = 50; ρ = 1.0 s^–1^, and τ_*e*_ = 1.3 ms. **(B)** Elimination of facilitation by a briefer τ_*e*_ = 0.3 ms coupled to the α transitions of the third conditioning impulse simulates the presence of intracellular calcium chelator in crayfish neuromuscular junction (Kamiya and Zucker, 1994). The inset shows persistence of asynchronous release with lower frequency after the third impulse.

A long τ_*e*_ = 1.3 ms reproduced fairly enough facilitation on the train of impulses and depression on the test stimulus (Figure 6A). The quantal output, which is hard to estimate from experimental records, could be predicted by the model (Figure 6).

It is worth to underscore that the same kinetic parameters that reproduce spontaneous and evoked release in previous sections here reproduced the facilitation–depression balance. Moreover, our simulations unexpectedly reproduced asynchronous release after the bulk of evoked release (inset in Figure 6A) in neuromuscular junctions of frog and fish (Miledi, 1966; Wen et al., 2010) and in other peripheral and central synapses (Zengel et al., 1980; Goda and Stevens, 1994; Atluri and Regehr, 1998; Best and Regehr, 2009).

The loss of facilitation by the sudden release of calcium chelator in crayfish presynaptic terminal (Kamiya and Zucker, 1994) was simulated by reducing τ_*e*_ in the third conditioning stimulus. Figure 6B shows that a τ_*e*_ = 0.3 ms value returned transmission to baseline and reduced depression upon the test pulse. Lower frequency asynchronous release persisted after the third train, suggesting spontaneous occurrence of spontaneous fusion in an enhanced pool of primed vesicles.

### The Balance From Facilitation to Depression

**FIGURE 7:**
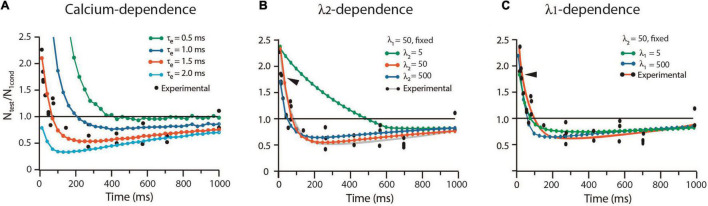
Calcium and rate constants influence short-term plasticity. **(A)** The duration of the calcium signal (τ_*e*_) determines the balance between facilitation and depression. **(B)** The λ_2_ coefficient determines the duration of facilitation. **(C)** The *λ_1_* coefficient reduces facilitation and depression. N_test_/N_1cond_ is the ratio between the amplitude of the response to the test pulse (N_test_) and the conditioned pulse (N_1cond_). Values above 1.0 indicate facilitation; values below 1.0 indicate depression. Experimental data obtained with license from Betz (1970).

The way by which the sequence of kinetic transitions affects the balance from facilitation and depression in frog was analyzed with the alternative protocol by Betz (1970). Experiments with high extracellular calcium concentration enhanced the release probability, while curare blocked acetylcholine receptors to render subthreshold transmission. Test impulses with different lags unveiled the time-dependence of depression. The long τ_*e*_ = 1.5 ms and our other frog parameters reproduced again the experimental results. As shown in Figure 7A, a briefer τ_*e*_ = 0.5 ms increased facilitation and abolished depression. By contrast, a longer τ_*e*_ = 2.0 ms eliminated facilitation but kept depression.

#### Effects of λ on Short-Term Plasticity

Contrary to the dominant effect of λ_1_ on low probability release, facilitation was dominated by λ_2_ (Figure 7B). A small λ_2_ = 5, which decelerates vesicle return to resting states, increased facilitation by 450% from 90 to 500 ms, without affecting its peak amplitude. However, large λ_1_ = λ_2_ = 500 values reduced and shortened facilitation (arrowheads in Figures 7B,C). Increasing or decreasing any λ coefficient reduced depression without affecting its time course (Figures 7B,C).

#### Vesicle Recycling Determines Short-Term Plasticity

**FIGURE 8:**
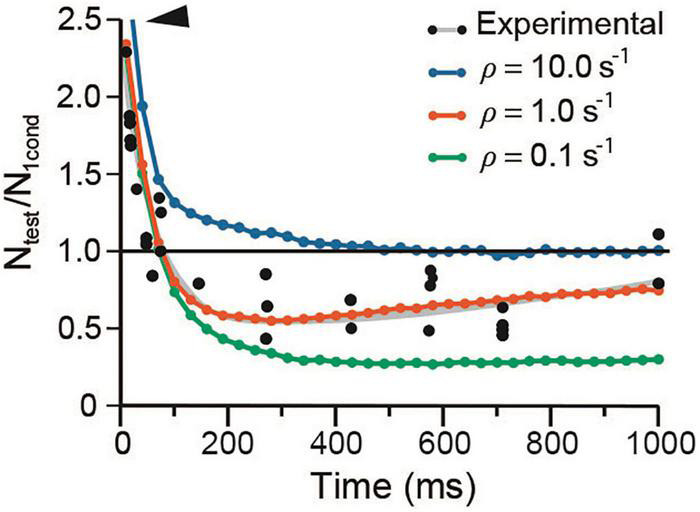
Vesicle recycling affects depression. The arrowhead denotes increased facilitation at high ρ value. Note the similar time course of the facilitation–depression sequence on extreme ρ values. Data were obtained with conventional α and λ values for frog.

It has long been hypothesized that depression occurs when the releasable-ready vesicle pool is reduced upon large release and slow recycling (Elmqvist and Quastel, 1965; Kusano and Landau, 1975). The mild effects of λ on depression in our simulations support such hypothesis. Figure 8 shows that a 10-fold acceleration of the mean recycling time (ρ = 10 s^–1^) while keeping λ_1_ = λ_2_ = 50, increased the amplitude and duration of facilitation and eliminated depression. Facilitation decayed biexponentially with a rapid τ_*e*_ = 30.19 ± 2.56 ms, followed by a slower τ_*e*_ = 169.55 ± 23.1 ms (*R*^2^ = 0.997). Conversely, a 10-fold reduction of ρ to slow down recycling did not affect facilitation, but increased depression from N_test_/N_1cond_ = 0.25 in the experimental data to a sustained 0.6 value by 450 ms.

#### Effect of the Number of Kinetic Steps on Short-Term Plasticity

**FIGURE 9:**
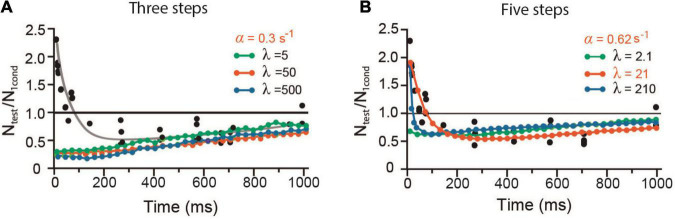
Short-term plasticity with different numbers of kinetic steps. **(A)** Three kinetic steps produced depression without facilitation. **(B)** Five kinetic steps reproduced short-term plasticity by using a larger α = 0.62 s^– 1^ and a smaller λ = 21.

The three-step model fed with the regular parameters of frog experiments or after 10-fold variations in their values failed to reproduce facilitation but maintained depression levels similar to those already described (Figure 9A). By contrast, a five-step kinetic model by the addition of a *D* state (Figure 9B) reproduced short-term plasticity, provided a larger α = 0.62 s^–1^ (as in mammalian neuromuscular junction), and a reduced λ = 21 for a β = value of 13 s^–1^. Depression was less susceptible to λ variations.

A six-step model also reproduced the experimental data provided an even larger α = 1.43 s^–1^ and smaller λ = 13, for β = 9.5 s^–1^. Such results support that the four-state kinetic sequence fed with one common set of parameters is necessary and sufficient to reproduce the dynamics of release from spontaneous to short-term plasticity.

### Activity-Dependent Dynamics of the Vesicle Pool

**FIGURE 10:**
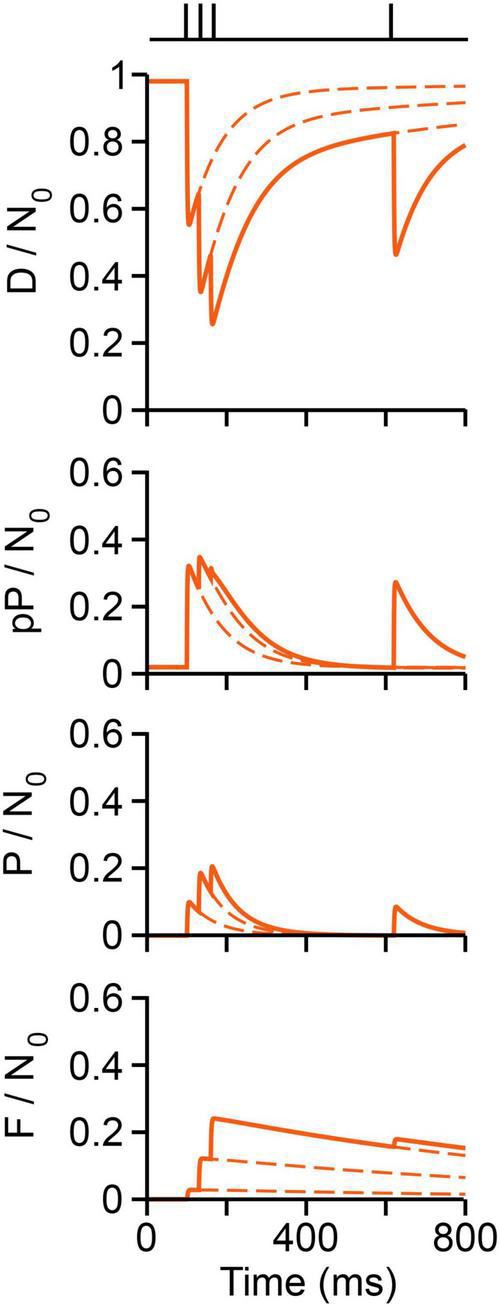
Vesicle dynamics in frog neuromuscular junction. The stimulation protocol is shown above (Mallart and Martin, 1968). The proportion of vesicles in each state is normalized to a pool of 10,000 (N_0_) vesicles. Calcium produces rapid *D ⇀ pP ⇀ P ⇀ F* transitions. Reversibility contributes to rapid recovery to resting state. Recycling contributes to slow recovery and depression.

The short-term plastic dynamics of transmission upon a conditioning train followed by a test pulse are plotted in Figure 10, following the experiment by Mallart and Martin (1968, see Figure 5). The fraction of vesicles in each state was normalized to *N*_0_ = 10,000. At rest, ∼98% vesicles are docked and the remaining 2% are decreasingly distributed in preprimed and primed states. About 300 vesicles (3%) fuse on the first impulse, as estimated by Katz and Miledi (1979), at 6°C. Therefore, ∼66% of vesicles that fuse were primed, the remaining arriving from immature states. Arrival of a second impulse encounters an increased population of preprimed and primed vesicles, thus evoking facilitation plus additional forward transitions in immature vesicles. After the third conditioning pulse, ∼25% of the total vesicle pool has fused. Such large release along with the slow recycling (F/N_0_ panel in Figure 10) depress the response to the test impulse (Figure 10).

## Discussion

Spontaneous release, evoked release, and short-term plasticity were reproduced here by a mathematical model of vesicles bound to a dynamic molecular fusion complex with four kinetic states. Our model provides a unifying mechanistic interpretation to the activity-dependent forms of release in a homogeneous vesicle pool. The backward rate constant and the much smaller forward rate constant values produce a vast majority of vesicles to rest in the docked state. Spontaneous and asynchronous fusion reflect spontaneous *P → F* occurrence in primed vesicles. The pattern of electrical activity determines the momentary proportion of vesicles in each maturation state. The model also predicts that the duration of facilitation depends largely on backward kinetic transitions, with increasing contribution of the recycling time constant as the number of conditioning impulses increases. The duration of depression reflects slow vesicle recycling.

### Multivariable-Dependence of the Frequency-Response of Release

The similar rate constants of the forward transitions in the fusion complex (Li et al., 2007; Chapman, 2008; Sudhof and Rothman, 2009) requires three additions to reproduce the whole dynamics of transmission. First, the calcium-dependence of every forward transition. Second, the calcium-independent backward *D* ↼ *pP* ↼ *P* transitions become synchronized by spontaneously following the highly synchronic forward transitions. Third, a minimum of four-transitions is necessary and sufficient to reproduce the whole dynamics of neuromuscular transmission studied here.

### Advantages of Four Over Three Transitions

It is interesting to note that the *pP* state buffers the effects of having logarithmic differences between the numbers of *D* and *P* vesicles. In absence of such buffering, a three-state sequence such as that suggested for the calix of Held synapse (Neher and Brose, 2018) results in exceedingly large amounts of release per impulse (Figure 2D). However, with adequate numbers of vesicles and release probabilities, the three-state sequence may reproduce the characteristic depression in the calix of Held (for review see von Gersdorff and Borst, 2002; Neher and Brose, 2018).

### Timing of Facilitation and Depression

The balance between the forward and backward transitions explains the frequency-dependent non-linear fluctuations of the quantal output during facilitation and depression. The sequential transitions in the fusion complex on an impulse increase largely the pool of primed vesicles after synchronous exocytosis, producing facilitation upon rapid arrival of another impulse. Vesicle priming after the impulse is predicted by the model from the decreased or increased fusion latencies when kinetic steps are reduced or increased, respectively (Figure 3D). A corollary to this observation is that the whole essence for facilitation is that the forward *D ⇀ pP ⇀ P* reactions continue after the synchronous release, producing a transient accumulation of newly primed vesicles. Without such possibility, transmission would be dominated by depression.

### Calcium Sensors in Each Transition Contribute to All Forms of Release

Our data suggest that one calcium sensor may produce fusion in all forms of release. This result seems to contradict the generally accepted contribution of at least two calcium sensors with different calcium affinities (Yamada and Zucker, 1992; Kamiya and Zucker, 1994; for review see Zucker and Regehr (2002); Sun et al. (2007)) and different forms of synaptotagmin controlling vesicle fusion in the neuromuscular junctions (Pang et al., 2006; Wen et al., 2010) and central synapses (Sudhof, 2013; Kaeser and Regehr, 2014; Kavalali, 2015; Volynski and Krishnakumar, 2018). However, our model predicts that the calcium sensors promoting each transition on an impulse contribute to modulate the dynamics of release.

Central synaptic vesicles seem to carry different types of synaptotagmin (Jahn and Südhof, 1994; Takamori et al., 2006). While fast synchronous release is produced by the activation of synaptotagmins 1, 2, or 9 (Chapman, 2002; Pang et al., 2006; Xu et al., 2007, for review see Kaeser and Regehr (2014), Neher and Brose (2018)), asynchronous release is supposed to depend predominantly on the high calcium affinity synaptotagmin 7 (Wen et al., 2010; Bacaj et al., 2013, 2015; Turecek and Regehr, 2018). Accordingly, theoretical models of transmission with two or three calcium sensors reproduce well the electrophysiological data (Goda and Stevens, 1994; Dutta Roy et al., 2014). For convenience, it is useful to focus this section by analyzing first the evidence concerning asynchronous release.

Evidence has long suggested that facilitation and asynchronous neuromuscular release rely on the exact same mechanism (Rahamimoff and Yaari, 1973; Zucker, 1996). Our simulations are consistent with this idea. The generation of asynchronous mepp_*s*_ using a reduced τ_*e*_ value to eliminate the residual calcium effect on release suggests that asynchronous release is an exacerbated version of spontaneous release with increased numbers of primed vesicles after a conditioning impulse. Other line of evidence suggests that synaptotagmin 7 drives asynchronous release (Wen et al., 2010; Bacaj et al., 2013; Turecek and Regehr, 2018), although evidence has also shown that the same vesicles may participate on both modes of release (Grigoryev and Zefirov, 2015). However, in neuromuscular junction of zebra fish, elimination of synaptotagmin 7 reduces but does not abolish asynchronous release (Wen et al., 2010). Therefore, both, spontaneous fusion and synaptotagmin 7-driven fusion may contribute to asynchronous release in the neuromuscular junction. The question is when does synaptotagmin 7 produce its effects. According to our simulations, synaptotagmin 7 may have its effects on the calcium-dependent maturation steps rather than producing vesicle fusion. Such statement is supported by diverse effects of synaptotagmin stabilizing the *D* state and to the maturation of the vesicle fusion complex (Reist et al., 1998; Loewen et al., 2006; Mohrmann et al., 2013; for review see Bowers and Reist (2020)).

### Relationship Between Facilitation and Asynchronous Release

The residual calcium hypothesis for paired pulse facilitation by Katz and Miledi (1968) and the third or fourth order calcium-dependence of release (Dodge and Rahamimoff, 1967; Smith et al., 1985; Augustine and Charlton, 1986) predict that low residual calcium levels activate high-affinity calcium sensors to produce supralinear vesicle fusion in facilitation (Zucker and Lara-Estrella, 1983; Yamada and Zucker, 1992; Van der Kloot and Molgó, 1993; Vyshedskiy and Lin, 1997; Zucker and Regehr, 2002; Ma et al., 2015). Our model suggests the possibility that the calcium sensors producing facilitation are those activating the *D ⇀ pP ⇀ P* transitions, which increase the pool of vesicles ready for release. Synaptotagmin 7 has emerged again as a candidate in central synapses (Sugita et al., 2001; Bacaj et al., 2013, 2015; Jackman and Regehr, 2017; Turecek and Regehr, 2018). However, as mentioned above synaptotagmin 7 may be acting on the early molecular transitions. Therefore, according to our model, fusion is produced by one calcium sensor, while the modulation of the number of vesicles that fuse depends on the action of the calcium sensors on the early transition states with synaptotaagmin 7 being one such sensors.

Electron tomography shows that from the moment of docking, the fusion complex has formed intimate boundaries with calcium channels (Harlow et al., 2001; Nagwaney et al., 2009; Szule et al., 2012). The interactions between fusion complex proteins and calcium channels have been analyzed in detail (for review see Catterall et al., 2013; Gandini and Zamponi, 2021). Such configuration may permit calcium sensors to catalyze every kinetic transition, as opposed to central synapses in which calcium channels may be separated from fusion complexes in immature vesicles (Neher, 2015). Other proteins thought to be involved in docking and priming such as RIM, Munc13, rabphilin, and Bassoon/Piccolo, have calcium-binding domains which may contribute to these transitions (Friedrich et al., 2010; Nishimune, 2012; Gundelfinger et al., 2016; Lai et al., 2017).

### Recycling and Depression

Our results confirm the essential role of vesicle recycling on depression and predict that backward transitions contribute to the amplitude of depression. Two or more recycling modes in the neuromuscular junction (Rizzoli and Betz, 2005) and central synapses (Wu and Borst, 1999; Sakaba and Neher, 2001; Schneggenburger et al., 2002) suggest equal numbers of recycling vesicle pools (for review see Alabi and Tsien, 2012). However, with a single recycling rate constant, our model reproduced convincingly the balance between facilitation and depression as studied by Betz (1970). However, we cannot exclude that the slow time constant of recycling in our model is masking faster events including some displaying a calcium-dependence (Sakaba and Neher, 2001).

## Materials and Methods

### Design of the Mathematical Model

The four-state kinetic model with six kinetic transitions shown in Figure 1 is the basis to analyze the collective behavior of a pool of 10,000 identical vesicles (Rizzoli and Betz, 2005). Six *R*_*j*_ transitions correspond to those in Figure 1, with *j* being a stochastic discrete variable with values *j* = 1, 2,…6, that correspond to each kinetic transition. Each transition occurs with an equal probability *a*_*j*_(*x*). The term *a*_*j*_(*x*)*dt* is the probability that an *R_j_* transition will occur in an infinitesimal time interval *t + dt*, when the system is in a state *X(t) = (D(t), pP(t), P(t), F(t)) = x*. Each *R_j_* transition is characterized by two quantities: One is the system state *x* = *D*(*t*),*pP*(*t*),*P*(*t*),*F*(*t*), which reflects the number of vesicles at each kinetic state. The second quantity is the vector *V*_*j*_(*v_D_j__*, *v_pP_j__*, *v_P_j__*, *v_F_j__*), which represents the change in the total number of vesicles over time at each state. At rest, a vast majority of vesicles lay in the *D* state. The effect of larger numbers of molecular states on transmission was analyzed by adding states with corresponding bidirectional rate constants between the *D* and *pP* states. In the three-state model the *pP* state was eliminated.

The stochastic kinetic model considers that fusion requires vesicles to arrive at the *P* state. Since the classical kinetic differential equations do not describe correctly the collective kinetics of a small number of vesicles (∼10,000 as compared to Avogadro’s number), we used instead the master Equation 1 for the probability distribution *P*(*x*, *t*; *x*_0_, *t*_0_) (Gillespie, 1976), whose solution describes the temporal evolution of the six transition probabilities between kinetic states. The rate constants are conventional probabilities per time unit (Gillespie, 1992):


(1) $$\frac{\partial P\left(x,t;x_{o},t_{o}\right)}{\partial t}=\sum_{j=1}^{6}\left(a_{j}\left(x-v_{j}\right)P\left(x-v_{j},t|x_{0},t_{0}\right)-a_{j}\left(x\right)P\left(x,t|x_{0},t_{0}\right)\right)$$

The solution of Equation 1 was simulated using the Gillespie algorithm (Gillespie, 1976), which emulates random transitions connecting different *X*(*t*) states. The fundamental equation of the Gillespie algorithm for the time evolution of the system is:


(2) $$p\left(j,\tau \right)d\tau =a_{j}\left(x\right)\exp\left[-\sum_{j=1}^{6}a_{j}\left(x\right)\tau \right]d\tau$$

Equation 2 predicts the probability that at a state *X*(*t*) = *x*, the next kinetic transition *R_j_*, will occur at the next infinitesimal time [*t* + τ, *t* + τ + *d*τ]. The random continuous variable τ advances the time in the simulations by the amount:


(3) $$\tau =-\left[\frac{1}{\sum_{j=1}^{6}a_{j}\left(x\right)\tau }\right]\text{ln}\left(r_{1}\right)$$

with *r*_1_ being a random number distributed uniformly in the interval (0, 1).

The probability distribution *p*(*j*, τ) *d*τ mimics the solution of the stochastic kinetic Equation 1 and plays a key role in the implementation of the stochastic algorithm. Thus, the random trajectories that connect different kinetic states, *X*(*t*) = *x*, describe the kinetic evolution of the vesicle pool.

The algorithm for the kinetic sequence can be summarized as follows: (1) The simulation begins by setting the initial state of the system *X*_*o*_ at time *t*_*o*_. (2) The propension functions *a*_*j*_(*x*) and their sum *a*_*o*_(*x*) = Σ*a*_*j*_(*x*) are calculated for each different time *t*. (3) The values of the discrete random variables *j* is chosen as the smallest integer that satisfies, $\sum_{k=1}^{j}a_{k}\left(x\right)>r_{2}a_{o}$ with *r*_2_ a random number distributed uniformly in the interval [0,1]. The continuous random variable τ is generated by applying Equation 3. (4) The transition to the next kinetic state *x → x + v_*j*_* and the time shifts to *t → t + τ* are calculated. (5) A new state (*x*, *t*) is obtained, and the procedure returns to step (1).

The simulation starts with *No* = 10,000 vesicles accumulated in the *D* state. In such conditions *X(t = 0) = Xo = (D(t = 0) = No*, and *pP(t = 0) = 0, P(t = 0) = 0, F(t = 0) = 0).* As the simulation progresses, the distribution of vesicles among the different states becomes stationary in about 5 min of the simulation. After this time our measurements in the simulations are made.

### Estimates of Kinetic Values

The activation energies involved in the molecular transitions from docking to exocytosis lay in the same order of magnitude (Li et al., 2007; Sudhof and Rothman, 2009). Therefore, we initially considered that α_1_ = α_2_ = α_3_ = α, and β_1_ = β_2_ = β. This strategy proved successful for reproducing every release mode. The α value used in cat simulations was estimated from the frequency distribution of spontaneous miniature potentials (Boyd and Martin, 1956a,b). The β and ρ values were fitted independently. Once adequate fittings were obtained, the variable values were evaluated within two logarithmic units. The model was simplified by using the coefficient λ = β/α, which permitted to evaluate the kinetic behavior in terms of the relative magnitudes of α and β. The code used in this study is available in the following repository: https://github.com/alexini-mv/kinetic-neurotransmission.

### Modeling the Calcium-Dependence

Presynaptic calcium elevations upon brief depolarization were modeled by adding a function *f*(*t*) to the forward rate constants, which acquired the form α_s_ = α + *f*(*t*). The kinetics of the calcium current decay in squid giant synapse experiments (Llinás et al., 1981a,b) served as the baseline. The onset of calcium transient was considered as instantaneous for the calcium channels in presynaptic neuromuscular terminals that are tightly bound to the fusion complex (Harlow et al., 2001; Nagwaney et al., 2009). Adjustments in the amplitude (in arbitrary units) and decay time (ms) of the artificial calcium elevation rendered successful results.

For our simulations it was more convenient to express the decay time τ_*e*_ of the calcium elevation instead of the decay time of the current, since according to the residual calcium hypothesis (Katz and Miledi, 1968; Kamiya and Zucker, 1994; Matveev et al., 2006), it is the residual free intracellular calcium after the impulse that promotes facilitation. The decay time of the calcium elevation was defined as:


(4) $$f\left(t\right)=\left\{ \begin{array}{c} 0\;\mathrm{if}\;t<t_{s}\\ I_{e}\exp\left(-\frac{t-t_{s}}{\tau_{e}}\right)\;\mathrm{if}\;t\geq t_{s} \end{array}\right.$$

where *t*_*s*_ is the stimulation time. The τ_*e*_ value was adjusted for each experimental protocol in the range of 0.05–1.5 ms. Once adjusted, the parameters of the calcium signal remained the same for each experiment. Calcium currents in certain central synapses may facilitate or depress upon subsequent stimulation (Borst and Sakmann, 1998; Cuttle et al., 1998; Forsythe et al., 1998; Inchauspe et al., 2004; Ishikawa et al., 2005; Xu and Wu, 2005; Mochida et al., 2008). However, our model rendered accurate results without any such modulation.

### Software, Hardware, and Statistics

The simulations were made in a custom-designed code using Python 3. Calculations were carried out in a personal computer with an AMD Ryzen 5 2500U processor.

The Pearson significance was calculated using a routine of the software Mathematica: https://reference.wolfram.com/language/ref/DistributionFitTest.html.

## Data Availability Statement

The datasets presented in this study can be found in online repositories. The names of the repository/repositories and accession number(s) can be found below: https://github.com/alexini-mv/kinetic-neurotransmission.

## Author Contributions

GR-S and AM-V designed the mathematical procedures and carried out the programming and mathematical analyses. FD-M provided the original idea and physiological context, and wrote the manuscript. All authors contributed to the discussion.

## Conflict of Interest

The authors declare that the research was conducted in the absence of any commercial or financial relationships that could be construed as a potential conflict of interest.

## Publisher’s Note

All claims expressed in this article are solely those of the authors and do not necessarily represent those of their affiliated organizations, or those of the publisher, the editors and the reviewers. Any product that may be evaluated in this article, or claim that may be made by its manufacturer, is not guaranteed or endorsed by the publisher.
